# An Analysis of Metabolic Changes in the Retina and Retinal Pigment Epithelium of Aging Mice

**DOI:** 10.1167/iovs.62.14.20

**Published:** 2021-11-19

**Authors:** Kristine A. Tsantilas, Whitney M. Cleghorn, Celia M. Bisbach, Jeremy A. Whitson, Daniel T. Hass, Brian M. Robbings, Martin Sadilek, Jonathan D. Linton, Austin M. Rountree, Ana P. Valencia, Mariya T. Sweetwyne, Matthew D. Campbell, Huiliang Zhang, Connor S. R. Jankowski, Ian R. Sweet, David J. Marcinek, Peter S. Rabinovitch, James B. Hurley

**Affiliations:** 1 Department of Biochemistry, University of Washington, Seattle, Washington, United States; 2 Department of Biology, Davidson College, Davidson, North Carolina, United States; 3 UW Diabetes Institute, University of Washington, Seattle, Washington, United States; 4 Department of Chemistry, University of Washington, Seattle, Washington, United States; 5 Department of Radiology, University of Washington, Seattle, Washington, United States; 6 Department of Laboratory Medicine & Pathology, University of Washington, Seattle, Washington, United States; 7 Department of Pharmacology and Toxicology, College of Medicine, University of Arkansas for Medical Sciences, Little Rock, Arkansas, United States; 8 Department of Molecular Biology, Princeton University, Princeton, New Jersey, United States; 9 Lewis-Sigler Institute for Integrative Genomics, Princeton University, Princeton, New Jersey, United States; 10 Department of Ophthalmology, University of Washington, Seattle, Washington, United States

**Keywords:** aging, retina, retinal pigment epithelium, metabolism, electroretinogram

## Abstract

**Purpose:**

The purpose of this study was to present our hypothesis that aging alters metabolic function in ocular tissues. We tested the hypothesis by measuring metabolism in aged murine tissues alongside retinal responses to light.

**Methods:**

Scotopic and photopic electroretinogram (ERG) responses in young (3–6 months) and aged (23–26 months) C57Bl/6J mice were recorded. Metabolic flux in retina and eyecup explants was quantified using U-^13^C-glucose or U-^13^C-glutamine with gas chromatography-mass spectrometry (GC-MS), O_2_ consumption rate (OCR) in a perifusion apparatus, and quantifying adenosine triphosphatase (ATP) with a bioluminescence assay.

**Results:**

Scotopic and photopic ERG responses were reduced in aged mice. Glucose metabolism, glutamine metabolism, OCR, and ATP pools in retinal explants were mostly unaffected in aged mice. In eyecups, glutamine usage in the Krebs Cycle decreased while glucose metabolism, OCR, and ATP pools remained stable.

**Conclusions:**

Our examination of metabolism showed negligible impact of age on retina and an impairment of glutamine anaplerosis in eyecups. The metabolic stability of these tissues ex vivo suggests age-related metabolic alterations may not be intrinsic. Future experiments should focus on determining whether external factors including nutrient supply, oxygen availability, or structural changes influence ocular metabolism in vivo.

Aging is associated with vision loss and physiological changes in ocular tissues.^1^^–^^4^ The retinal pigment epithelium (RPE) accumulates lipofuscin and drusen, Bruch's membrane thickens, and cellular organization decreases.^5^^–^^10^ These alterations are also characteristic of age-related macular degeneration, a severe age-associated disease that is a leading cause of vision loss.^2^^,^^11^^–^^14^ With age, the neural retina thins,^15^^–^^17^ synaptic connections deteriorate,^16^^,^^17^ photoreceptor function declines,^18^^,^^19^ and their mitochondria are damaged.^20^^–^^22^

The amplitude of scotopic and photopic electroretinograms (ERGs) are reduced,^18^^,^^23^^–^^26^ and both optokinetic tracking^27^^,^^28^ and visual discrimination tasks^28^^,^^29^ suggest a reduction in spatial acuity and contrast sensitivity with aging. Retinal cells do not appear to be lost in significant number or are lost in selective areas of the retina^15^^,^^16^^,^^30^ with age and rhodopsin levels remaining stable,^31^ suggesting the function of existing cells decline due to changes in other cellular processes, such as decreased electrical resistance.^19^^,^^27^ We hypothesized that a reduction in metabolic function in retinal cells may be an additional factor that contributes to diminished retinal responses to light in aging. Manipulating metabolism ex vivo directly influences ERG response.^32^ Previous studies suggest metabolic interplay exists between the highly glycolytic retina and the RPE^33^^–^^35^ and that metabolic changes in the RPE can affect the retina. Increasing or disrupting glucose conduction in the RPE can cause retinal degeneration.^36^^–^^38^ Although steady-state metabolite levels differ in ocular tissues isolated from young and middle-aged mice,^23^ to our knowledge, the cross-talk between aging and central energy metabolism in the mammalian retinal ecosystem has not been examined in advanced age. Based on pilot studies in our laboratory, we hypothesized aging would have contrary effects on the retina and RPE and impact function. We expected decreased glycolytic and TCA flux in retina with increased oxidative metabolism, and anticipated the opposite in the RPE with increased glycolytic flux and reduced mitochondrial metabolism. To investigate the relationship between retinal response to light and energy metabolism in aging retina and RPE (study structure in Fig. 1), we measured retinal function in vivo by ERG and interrogated intrinsic alterations of metabolism by analyzing ex vivo tissue explants using stable isotope tracers^39^ and targeted metabolomics.

**Figure 1.**
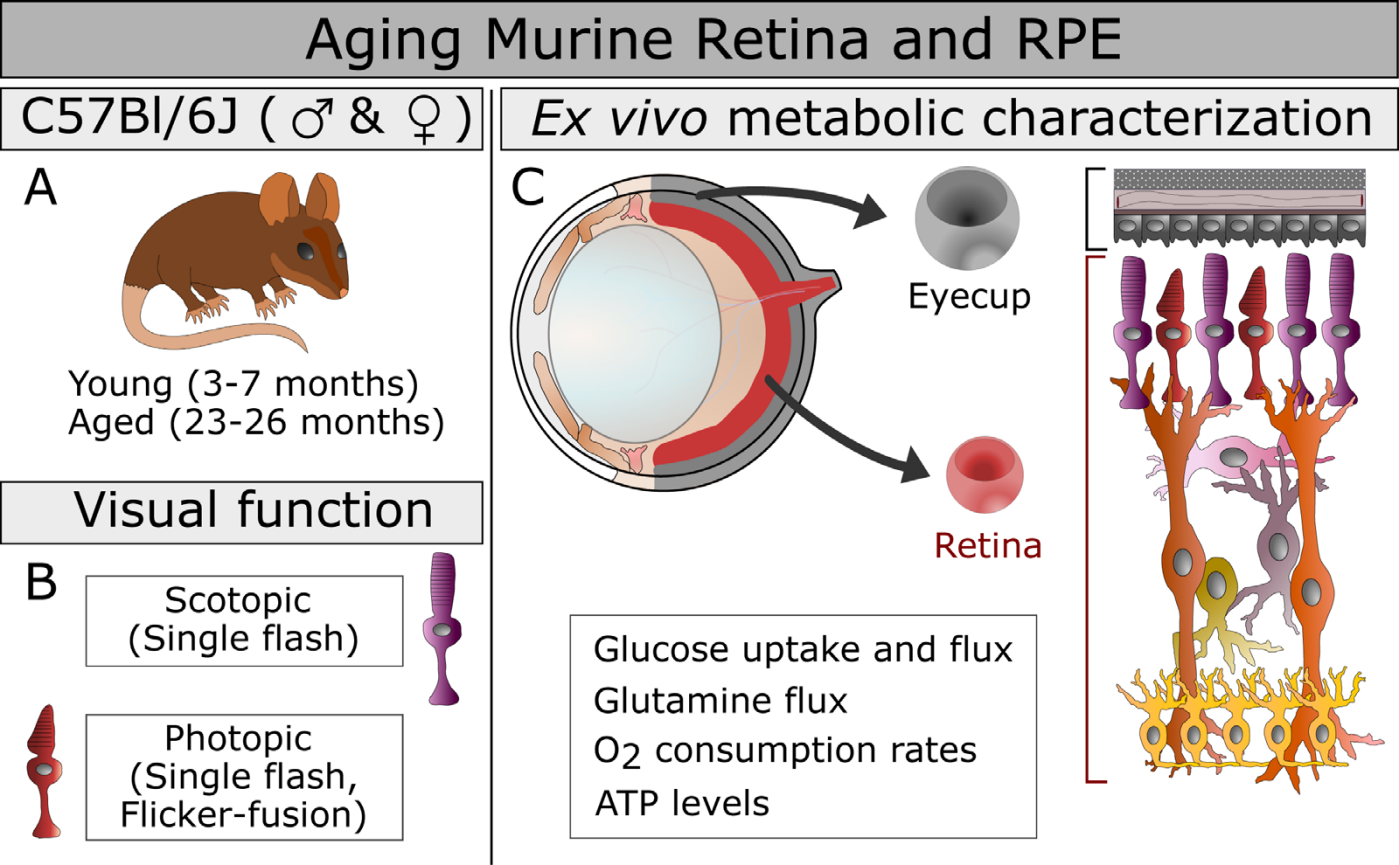
The study design is presented including the mouse groups, ages, and sex (**A**), the measures of visual function used (**B**), and the ex vivo approaches to characterizing metabolism (**C**) in the aging eye. The functional metabolic measurements are listed below the structure of the murine eye **C**, top left, from which we isolated two explants in this study **C**, center,: the retina RPE-choroid complex that includes the RPE, choroid, and sclera that has been cleared of connective tissue (eyecup). The cellular composition of these explants **C**, right, is highlighted with retinal cells shown in shades or red, purple, orange, and yellow, whereas the eyecup is shaded in variations of gray.

## Methods

### Mouse Ages, Origin, and Housing

C57Bl/6J mice of both sexes were obtained from the National Institute of Aging aged rodent colony (Bethesda, MD, USA) and Jackson Laboratories (Bar Harbor, ME, USA; STOCK NO: 000664). Young (3–7 months) and aged (23–26 months) mice were housed in groups of 5 or less with ad libitum food (Rodent Diet 5053) and water. Mice were confirmed free of the Crb1 mutation found in Rd8 models of retinal degeneration.^40^^,^^41^ The light/dark cycle was 14/10 hours. Some aged male mice were treated with saline via osmotic minipumps or provided water by bottle for 8 weeks prior to euthanasia as controls for separate studies unrelated to the eyes. No differences were observed in ocular metabolism between untreated and control mice. Experiments complied with the policies of the Animal Care and Use Committee at the University of Washington and the ARVO Statement for the Use of Animals in Ophthalmic and Vision Research.

### Electroretinogram Set-Up

Mice were dark adapted overnight (approximately 17 hours), anesthetized with isoflurane, and the eyes were dilated with 2.5% phenylephrine (Akorn, Inc; NDC 174780201-15) and 1% tropicamide (Bauch + Lomb; NDC 24208-585-64). Gold electrodes were placed on each cornea. A reference and ground electrode were positioned on the back of the head. Mice were placed inside a UTAS Visual Diagnostic System with BigShot Ganzfeld with UBA-4200 amplifier (LKC Technologies, Gaithersburg, MD, USA). Readings were taken from both eyes, and the eye with the best response was considered. Scotopic assays were performed first, followed by photopic flicker or photopic single-flash experiments under a continuous 30 cd/m^2^ background light.

### Single-Flash ERGs

Recordings were elicited using flashes of LED white light at increasing intensities with 2-minute pauses between flashes under scotopic (−50 to 50 dB) and photopic (0 to 100 dB flashes, 30 cd/m^2^ background light) conditions. Readings were calibrated such that 0 db = 2.5 cd*s/m^2^. The a-wave amplitude was measured 8 ms after stimulus. The b-wave amplitude was measured as the magnitude from the a-wave minimum to the b-wave maximum.

### Flicker-Fusion ERGs

**Figure 2.**
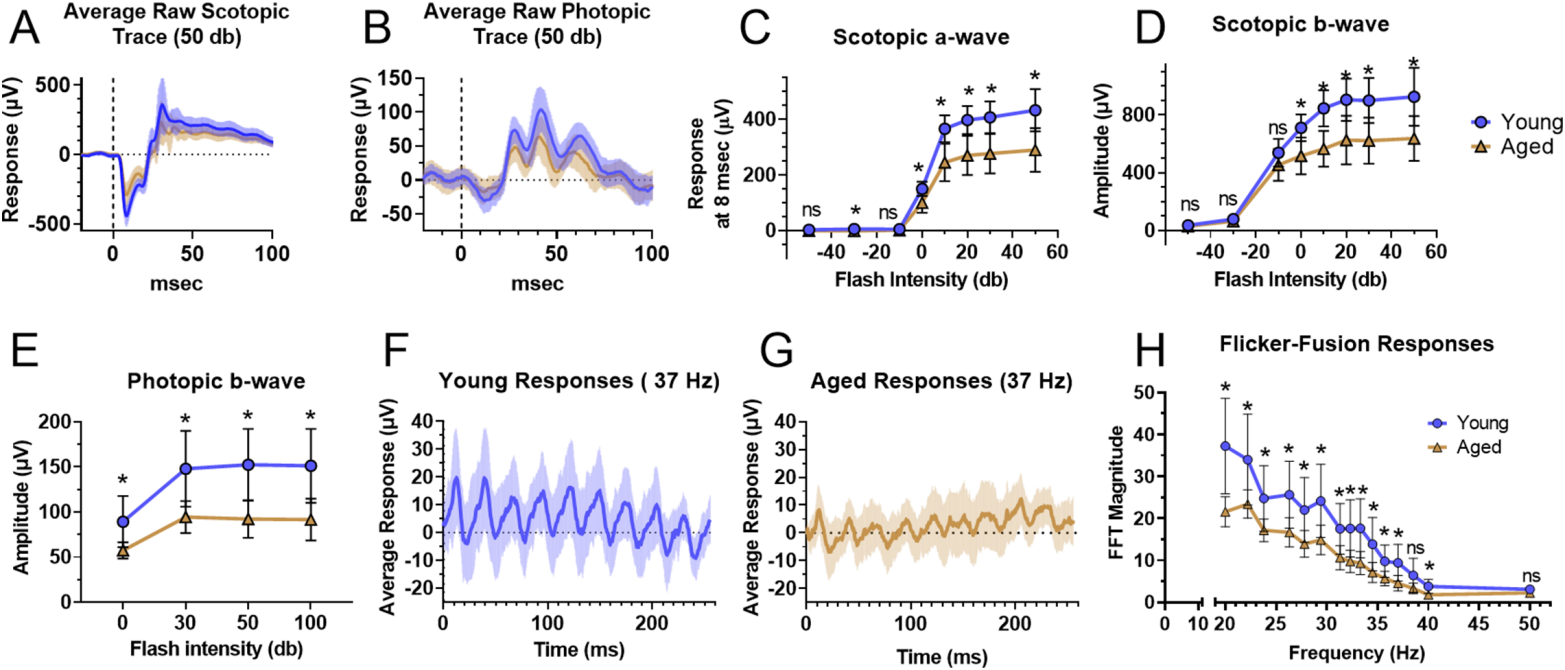
Single-flash ERGs were used to assess scotopic and photopic function in the retina. The averaged response curve in response to a 50 db flash are shown under scotopic (**A**) and photopic (**B**) conditions. Under scotopic conditions, the a-wave amplitude at 8 ms after the flash (**C**) and the b-wave amplitudes (**D**) were extracted from −50 to 50 db flashes of light. Both the a-wave and b-wave indicate a decline in the response of rod circuitry with aging. In photopic conditions (background light of 30 cd/m^2^), the average response curve to a 50 db flash **B** and the resulting b-wave magnitude from flashes of magnitude 0 to 100 db (**E**) show a decline in the response of cone circuitry with aging. Photopic function and temporal resolution were examined with a 5 db flash which flickered between frequencies of 20 to 50 Hz. The averaged response to 5 db light flickering at 37 Hz is shown for young (**F**) and aged animals (**G**). The young have a more uniform and stronger response to equivalent stimuli than aged animals. Aging decreased the magnitude of the response at equivalent frequencies (**H**) as calculated by Fast Fourier Transform. Sample sizes in scotopic single-flash and flicker measurements were 8 for young and 9 for aged, and in photopic single-flash measurements there were 12 young and 9 aged mice. Panels show the average ± standard deviation. Normality of data was determined using the Shapiro-Wilk test and *P* values were calculated using Mann-Whitney tests (* = *P* < 0.05).

Temporal resolution was measured under photopic conditions using a 5 db flash of varied frequencies (20–50 hertz [Hz]) with 10 second pauses between frequencies and 9 replicates. Measurements were taken at 0.5 second intervals for a total of 512 points in 0.255 seconds (sampling frequency = 2003.91 samples/second). At each frequency, replicates were averaged. Figures 2F and 2G show examples of these raw values at 37 Hz. A waveform was generated and the magnitude was calculated using Fast Fourier Transform (Microsoft Excel Data Analysis ToolPak; sampling frequency = 2003.9 samples/second, step value = 3.91).

### Isolation of Retinas and Eyecups

Animals were euthanized by awake cervical dislocation. Eyes were enucleated, cleared of excess tissue, and the retina separated from the RPE-choroid-sclera complex (eyecup) in Hanks’ Balanced Salt Solution (Gibco, Grand Island, NY, USA) within approximately 5 minutes. Tissues for ATP determination were snap frozen and stored in liquid nitrogen. Those for flux or O_2_ consumption rate (OCR) were used immediately.

### Metabolic Flux and Metabolite Extraction

Metabolite standards and buffer components were purchased from Sigma-Aldrich (Millipore Sigma, St. Louis, MO, USA). Product numbers are included in Supplementary Table S2. Incubation medium was formulated as follows:

• Krebs's Ringer Buffer (KRB): 98.5 mM NaCl, 4.9 mM KCl, 1.2 mM KH_2_PO_4_, 1.2 mM MgSO_4_, 20 mM HEPES, 2.6 mM CaCl, and 25.9 mM NaHCO_3_
• Either 5 mM U-^13^C glucose alone or 5 mM unlabeled glucose and 2 mM U-^13^C glutamine

U-^13^C metabolic tracers (99% isotopic purity) included ^13^C labeled D-Glucose and L-Glutamine (Cambridge Isotope Laboratories, Inc., Tewksbury, MA, USA). Incubations, tissue extractions, and media extractions were performed as described^39^ with minimal changes. Some samples were split in half by volume before drying.

### Gas Chromatography-Mass Spectrometry Analysis

Calibration curves were generated with metabolite standards (1.25–35 µM). Derivatization and selected ion monitoring (SIM) methods have been described previously^39^ with few changes. Incubation with N-tert-butyldimethylsilyl-N-methyltrifluoroacetamide (Millipore Sigma, St. Louis, MO, USA) was increased to 60 minutes. Samples were analyzed using a 49-minute gradient on an Agilent 7890/5975C gas chromatography-mass spectrometry (GC-MS) system. For all derivatized metabolites, target ions were used for quantification and isotopologue distribution, and a qualifier ion for identity confirmation. Supplementary Table S2 describes SIM method details. Peak areas were obtained in MSD Chemstation (Agilent Technologies, Santa Clara, CA, USA) with manual verification of integrations. IsoCor version 2^42^^,^^43^ corrected for natural ^13^C abundance and determined percent enrichment. In isotopologues, the number of incorporated ^13^C is represented shorthand by “Mx,” where x is the number of ^13^C (M0, M1, etc.).

### Oxygen Consumption Rate

Two retinas or four eyecups per replicate were quartered and loaded into a perifusion system that assesses OCR as described^44^^,^^45^ with minimal changes. Tissue was perifused with KRB, 0.1 g/100 mL BSA, 1X antibiotic-antimycotic (Gibco, Grand Island, NY, USA) and 5 mM glucose. An artificial lung maintained 21% O_2_, 5% CO_2_, and 74% N_2_. Succinate (5 mM) was added when testing mitochondrial function. OCR was calculated as the product of flow rate times the difference in outflow and inflow oxygen. Data were reported as a change in OCR after subtracting the baseline OCR in 5 mM glucose alone.

### ATP Measurements

ATP pools in retinas and eyecups extracted in boiling water^46^ were measured via luminescence with the Molecular Probes ATP Determination Kit, per the manufacturer's instructions.

### Protein Concentration for Normalization

Flux and ATP pools were normalized to protein content. Protein pellets were solubilized in RIPA buffer (150 mM NaCl, 1% Triton X-100, 0.5% sodium deoxycholate, 0.1% SDS, 50 mM Tris pH 8.0, and 1X HALT protease/phosphatase inhibitor) and quantified using the Pierce BCA Protein Assay Kit, per the manufacturer's instructions.

### Grouping and Statistics

Extractions, derivatization, and sample runs were processed in batches including ages, genders, matched retinas, eyecups, and/or their media, and random time points. To account for circadian contributions, the time of death for animals used in glucose flux, glutamine flux, and OCR are plotted in Supplementary Figure S1C. Sample sizes represent biological replicates from different animals. Normality was examined using the Shapiro-Wilk test. The majority of data was not normally distributed, thus nonparametric tests were used. Age differences were examined using Mann-Whitney tests. Changes associated with age and sex were considered using Kruskal-Wallis and Dunn's multiple comparison tests. Significant results (*P* < 0.05) are marked with “*” and comparisons that did not reach statistical significance are marked “ns”.

## Results

### Scotopic and Photopic ERG Responses are Diminished in Aging Mice

We recorded scotopic and photopic ERGs in young (4–5 months) and aged (26 months) male mice. The a-wave and b-wave amplitudes were extracted from raw traces of single flash scotopic ERGs (see Fig. 2A). The a-wave response measured 8 msec after the flash were significantly reduced between young and aged mice at −30 db and between 0 and 50 db (see Fig. 2C) and the b-wave amplitude was reduced from 0 to 50 db (see Fig. 2D). The scotopic a-wave and b-wave decreased proportionally with age. Cone-mediated retinal function was characterized using b-wave amplitudes in raw traces (see Fig. 2B) from single-flash photopic ERGs. The b-wave amplitude declined significantly in aged animals (see Fig. 2E). These findings correspond with previous studies where a- and b-waves were reduced in mice as early as 16 months.^23^^,^^24^

Temporal resolution was considered with flicker-fusion ERG using 5 db flashing light (20–50 Hz). An overall loss in temporal resolution was seen in the averaged raw responses. Young (see Fig. 2F) and aged (see Fig. 2G) averages are shown at 37 Hz. After Fast Fourier Transformation, the magnitude declined significantly with age at all frequencies except 38.5 and 50 Hz (see Fig. 2H).

### Metabolic Flux From Glucose Is Preserved in Retinal Explants Cultured Ex Vivo

**Figure 3.**
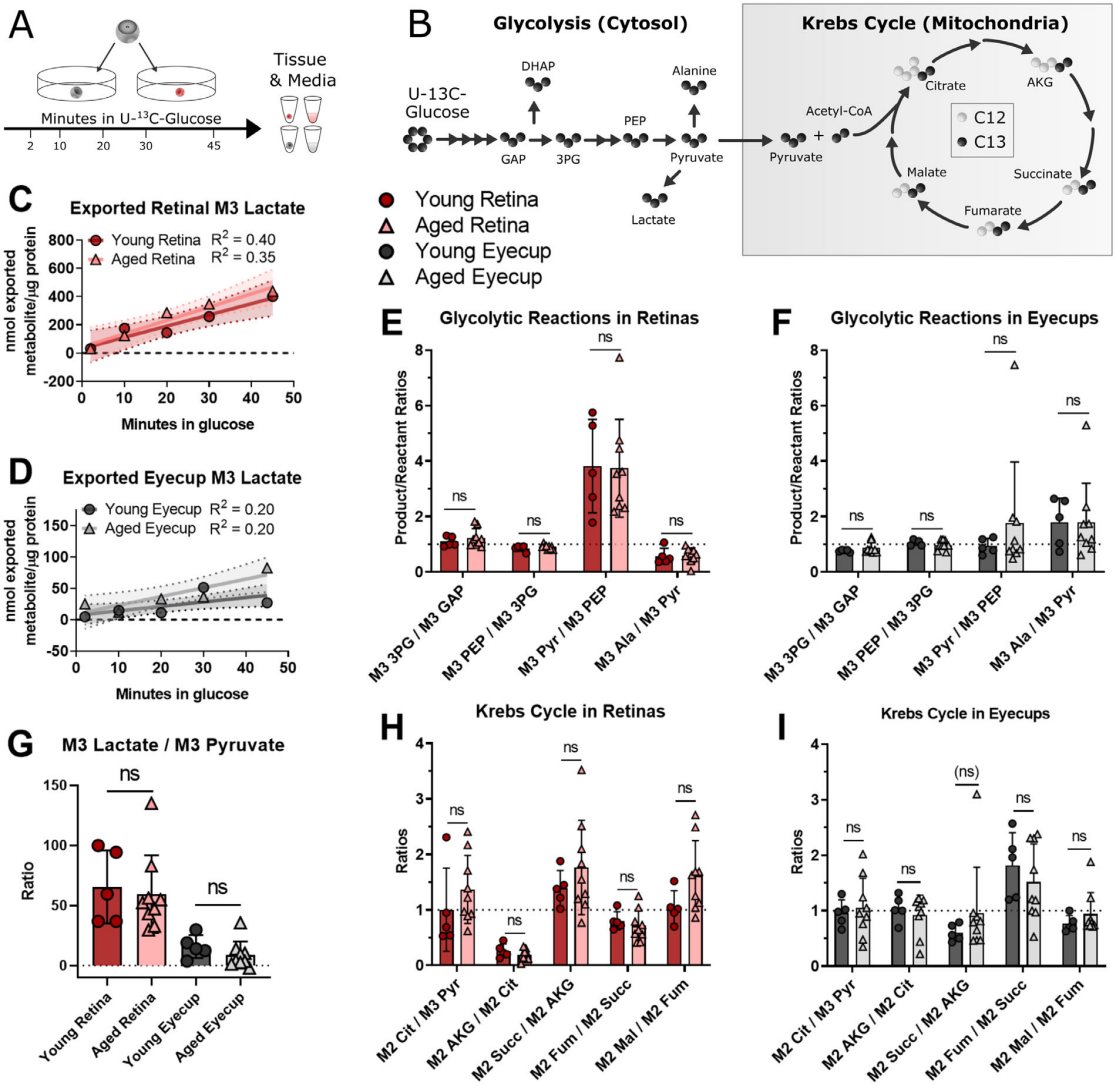
Metabolic activity was examined by incubating retinal and RPE-choroid (eyecup) explants in U-^13^C-glucose between 2 and 45 minutes. The tissue was washed and frozen, and aliquots of the incubation media were collected for analysis (**A**). Labeled intermediates downstream of glucose were quantified (**B**) in terms of percent ^13^C incorporation, pmol of ^13^C-labeled isotopologue per µg of protein in the retinal or eyecup explant, and product:reactant ratios for glycolytic reactions and those pathways that can be entered via pyruvate. Percent incorporation, pool sizes, and amount of isotopologue are shown in Supplementary Figures S3 and S4. To examine glycolytic activity, the amount of exported M3 lactate was measured in the incubation media of retinas (**C**) and eyecups (**D**). One aged eyecup at 30 minutes was found to be an outlier by Grubb's test (alpha = 0.05, *P* < 0.05) and removed. The slopes of the lines were all non-zero in retina (p_young_ = 0.002 and p_aged_ = 0.0003) and eyecups (p_young_ = 0.03 and p_aged_ = 0.009), but showed no significant age-related change (regression slope: p_retina_ = 0.7 and p_eyecup_ = 0.3). Within tissues, product:reactant ratios in glycolysis and common exit points to other pathways were plotted at 30 minutes because both tissues had reached a steady state. No significant age-related changes were seen in retina (**E, G**) or eyecup (**F, G**). Moving into the Krebs Cycle **B** at 30 minutes, no statistically significant age-related were observed in retina (**H**) or eyecup (**I**) explants. Normality of data was determined using the Shapiro-Wilk test and *P* values were calculated for age-related comparisons using Mann-Whitney tests (* = *P* < 0.05). Error bars represent the standard deviation, except in panels **C** and **D**, which show the 95% confidence interval for the linear regression. GAP, glyceraldehyde 3-phosphate; DHAP, dihydroxyacetone phosphate; 3PG, 3-phosphoglycerate; PEP, phosphoenolpyruvate; Pyr, pyruvate; Ala, alanine; Cit, citrate; AKG, α-ketoglutarate; Succ, succinate; Fum, fumarate; Mal, malate.

We sought to determine if retina and RPE glucose usage is reduced at an age with reduced ERG responses (see Fig. 2). Retinas and eyecups were isolated from young (3–5 months) and aged male (26 months) and female (23–25 months) mice. Labeled metabolites generated from U-^13^C-Glucose in tissues and incubation media were measured by GC-MS (Fig. 3A). Metabolites were quantified in terms of pmol or nmol metabolite per µg of protein. We examined metabolites in the tissue or exported by the tissue and considered the percent of the total pool labeled with ^13^C, and product:reactant ratios for particular steps in glycolysis and the TCA cycle.

Glycolytic intermediates (see Fig. 3B) in the explants were considered first. We found no consistent change in pool size, percent ^13^C-incorporation, and labeled isotopologues in either tissue (Supplementary Fig. S2). Export of M3 lactate from the retinas (see Fig. 3C) and eyecups (see Fig. 3D) into the incubation medium was stable. We examined product:reactant ratios at 30 minutes when the label incorporation had stabilized and found no substantial age-associated changes in glycolytic reactions in retinas (see Figs. 3E, 3G) or eyecups (see Figs. 3F, 3G). Glycolytic function appears preserved in both tissues.

Next, we considered how age impacts the Krebs cycle. As with glycolysis, we saw no consistent changes in retinas (Supplementary Fig. S3A–C) or eyecups (Supplementary Fig. S3D–F) in pool size, percent ^13^C incorporation, or labeled isotopologues. There were no significant age-related changes in product:reactant ratios in retinas (see Fig. 3H) or eyecups (see Fig. 3I) at the same 30-minute time point considered for glycolysis. Age did not significantly impact the Krebs cycle in retinas and eyecup explants.

Sexual dimorphism can influence outcomes in biological studies,^47^ and differentially impact the visual system generally^48^ and in aging.^49^^,^^50^ Glucose processing in young and aged female mice was considered in parallel to males at 2 minutes. Total protein content in retinas was unchanged between males and females (see Supplementary Fig. S1A), however, in the eyecups (see Supplementary Fig. S1B) there was a change in aged males accounted for by normalizing all values to tissue protein content. There were no significant contributions of age or sex in pool size or isotopologues (Supplementary Figs. S4A, S4B) in the retina or eyecups (see Supplementary Figs. S4C, S4D). No sex-dependent differences in aging retinal or eyecup metabolism were found.

### Glutamine Metabolism is Altered Ex Vivo in Aged Eyecups

Mitochondrial dysfunction is a hallmark of aging.^51^ To more directly investigate mitochondrial metabolism in aged ocular tissues we examined the usage of glutamine and succinate, which are oxidized by RPE mitochondria.^52^^,^^53^

**Figure 4.**
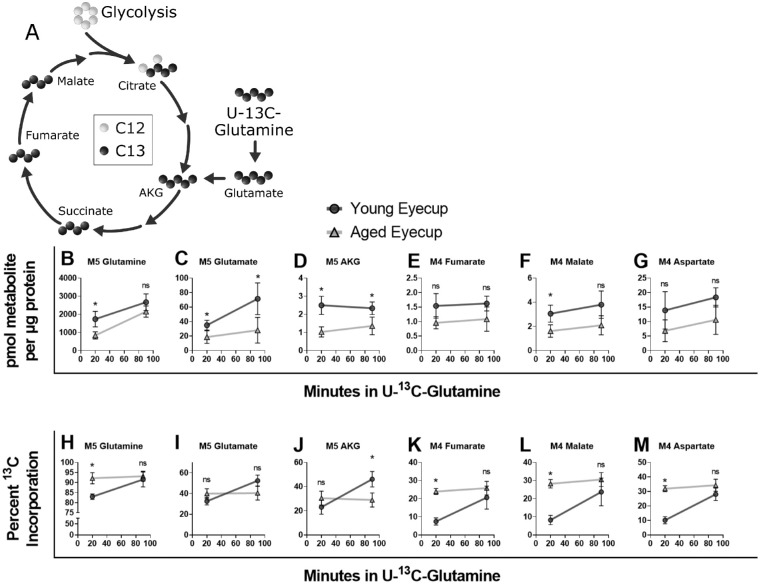
Metabolic activity with glutamine – and in the mitochondria more broadly – was examined by incubating eyecup explants in U-^13^C-glutamine for 20 or 90 minutes. The M4 and M5 labeled isotopologues entering into and proceeding through the Krebs cycle (**A**) were quantified in terms of pmol of ^13^C-labeled isotopologue per µg of protein in eyecup explants (**B–G**). M5 glutamine **B**, glutamate **C**, and AKG **D** trended lower in the aged eyecups compared to young at both time points examined, although glutamine did not reach statistical significance at 90 minutes. In the downstream intermediates fumarate **E**, malate **F**, and aspartate **G** this decline was reproduced, but did not reach statistical significance. The percent incorporation of ^13^C remains essentially unchanged in aged eyecups at both time points in all intermediates examined (**H–M**). Young percent ^13^C incorporation rises past the aged in glutamate **I** and AKG **J**. However, in glutamine **H**, fumarate **K**, malate **L**, and aspartate **M**, the percent of ^13^C incorporation was consistently lower in young eyecups at 20 minutes, but had essentially matched the aged by 90 minutes. These changes can be related back to pool size and product:reactant ratios, which are included in Supplementary Figure S5. Normality of data was determined using the Shapiro-Wilk test and *P* values were calculated for age-related comparisons using Mann-Whitney tests (* = *P* < 0.05). Error bars represent the standard deviation.

Glutamine usage in eyecup and retina explants was measured with ^12^C-glucose and U-^13^C-glutamine. Metabolite pool size was diminished in aged eyecups (Supplementary Fig. S5A) in glutamine, glutamate, and the downstream Krebs cycle metabolites α-ketoglutarate, fumarate, malate, and aspartate. Because we did not observe this decline in eyecups provided only glucose (see Fig. 3), we hypothesized that aged eyecups may have a defect in mitochondrial glutamine metabolism. After incubation in ^13^C-labeled glutamine, M5 glutamine, glutamate, and α-ketoglutarate (Fig. 4B–D) were significantly lower in aged eyecups. Levels of M4 Fumarate, M4 malate, and M4 aspartate (see Fig. 4E–G) trended lower in aged eyecups compared to young. The percent labeled with ^13^C (see Fig. 4H–M) varied by metabolite. Notably, M5 glutamine, M4 fumarate, M4 malate, and M4 aspartate were lower in young eyecups at 20 minutes, but essentially matched the aged by 90 minutes. The product:reactant ratio of M5 glutamate/M5 glutamine (see Supplementary Fig. S5B) decreased with age. These data suggest glutamine anaplerosis is diminished in aged eyecups.

The same analysis was performed in retinas. In contrast, no significant age-related changes in pool size or ^13^C-labeled intermediates (see Supplementary Figs. S6A, S6C) were observed. Percent incorporation at 20 minutes was higher in later Krebs cycle intermediates of aged retinas, but younger retina label uptake nearly matched aged by 90 minutes (see Supplementary Fig. S6B), although it was still lower. No significant changes in product:reactant ratios (see Supplementary Fig. S6D) were observed.

We postulated the decline in eyecup glutamine metabolism was suggestive of reduced mitochondrial function, which we approximated by measuring OCR and ATP levels in young and old retinas and eyecups. Succinate is a mitochondrial respiratory substrate preferentially oxidized by the RPE.^52^ Succinate-driven OCR was calculated for each animal by taking the maximum steady-state OCR of tissue respiring on 5 mM glucose + 5 mM succinate and subtracting the 5 mM glucose baseline OCR (Supplementary Fig. S7A) and retinas (see Supplementary Fig. S7B). Steady-state OCR values are listed in Supplementary Table S1. As reported previously, succinate stimulates OCR in eyecups to a significantly greater degree compared to the retina.^52^ Neither retinas nor eyecups exhibited significant age-related changes in succinate-stimulated O_2_ consumption (see Supplementary Fig. S7C, S7D). Steady-state levels of ATP (see Supplementary Fig. S7E) also showed negligible changes.

## Discussion

Aging reduces retinal ERG response in humans^25^^,^^26^ and mice.^18^^,^^19^^,^^23^^,^^24^ We sought to examine responses in 26 month old C57Bl/6J mice, at such time that about 25% of the population would have died^54^^,^^55^ and are roughly equivalent to 79 year old humans.^56^ Previous trends were confirmed – photopic temporal resolution and both rod and cone electrical responses to light declined relative to young mice (see Fig. 2). Although there are confounding factors associated with the measurement of the ERG, other work utilizing optokinetic tracking and visual discrimination tasks found diminished visual acuity.^27^^–^^29^ We hypothesized this diminished response would be concurrent with reduced glucose metabolism and mitochondrial function in aged retina and eyecup explants.

Rapid aerobic glycolysis in the retina is required for visual function.^32^^,^^34^ Despite reduced ERG responses, retinal glucose and glutamine metabolism is stable ex vivo. Both glycolytic and Krebs cycle intermediates (see Fig. 3, Supplementary Figs. S2, S3) were essentially unchanged between young and aged retinas. Although glycolysis had reached a steady-state and thus we could not measure flux within tissues, the ^13^C lactate exported into culture medium can approximate the rate of glycolysis. This complementary measurement was also unchanged with age. There was no observed effect of sex (Supplementary Fig. S4), and mitochondrial OCR and ATP levels were also unchanged. Glutamine metabolism is linked to synaptic transmission through the neurotransmitter glutamate^57^ and additional experiments will be required to determine if the small differences with aging in retinas (see Supplementary Fig. S6) translate to changes in neurotransmission. We postulate that aging retina is robust and may retain metabolic function on par with young retinas despite significant physiological alterations in vivo.

Glucose metabolism appears to be preserved in aging eyecups (see Fig. 3, Supplementary Figs. S3, S4). However, glutamine metabolism in aged eyecups differs from young. Although our sample size (*n* = 4–5) leaves subtle differences challenging to identify, pool size (see Supplementary Fig. S5) and isotopologues decreased with age. However, the percent of ^13^C incorporation into glutamine intermediates (see Fig. 4) remained stable in aging eyecups compared to the increasing percent in young eyecups. We hypothesize this indicates aged eyecups are deficient in glutamine metabolism and reach their maximum metabolic capacity faster than their younger counterparts. While the decreased M5 glutamate:M5 glutamine ratio could be indicative of decreased glutaminase activity in aging RPE, enzyme activity assays from glutaminase to fumarase would allow more targeted identification of the source of the defect in glutamine metabolism that we observed.

Finally, we considered that mitochondria have diverse functions outside cellular respiration.^58^^–^^60^ Abundance of Krebs cycle intermediates provide a direct link to epigenetic changes.^61^^,^^62^ Mitochondrial dysfunction is also involved in cellular signaling and aging having broad implications in an organ system.^51^^,^^59^^,^^63^^–^^66^ Although the ocular tissues we examined – especially retina – may be resilient to age-related metabolic defects, future research will be critical to clarify how other functions of mitochondria in retina and RPE influence the broader ocular ecosystem in aging.

### Study Limitations and Considerations for Future Studies

That glucose metabolism was relatively robust with age was unexpected compared with other aged tissues. Metabolic changes and mitochondrial dysfunction are hallmarks of aging.^51^ Glycolytic capacity declines in the aged brain and effector T cells.^67^^–^^70^ Young hearts subsist on fatty acid oxidation, but switch to glycolysis and ketone bodies in aging and heart failure.^71^^,^^72^ Liver and skeletal muscle experience metabolic shifts.^73^^–^^78^

Our experimental design and technique influenced our findings. Subsequent experiments in our laboratory^52^ have suggested glycolytic flux is best examined in earlier time points. Although we can approximate glycolytic flux in this study by examining lactate export in the culture medium (see Figs. 3C, 3D), a direct examination of time points of 2 minutes and below will be necessary. The explants in our analyses were exposed to physiological levels of glucose, but oxygen levels were nonphysiological and, unlike conditions within an eye, there were few barriers to transport. Blood vessel function around the retina declines in aging people^79^^–^^81^ and O_2_ extraction is lower in aged human retina,^82^ which may indicate that nutrient and O_2_ access is restricted in an aged mouse. Aged retina and eyecup appear to maintain most of their metabolic function ex vivo. However, in the eye of an aged animal, they may experience non-cell autonomous dysregulation of metabolism. Previous studies^83^^,^^84^ using this ex vivo approach identified intrinsic, cell autonomous differences between the groups.

An in vivo analysis of metabolic flux will be necessary to establish whether metabolism is altered within the aging eye, and whether it would be due solely to intrinsic properties of the tissues or related to nutrient access. Direct infusion of ^13^C-labeled fuels^85^ would be an effective way to test this. The analyses described in this report can be a starting point to a more physiological assessment of glycolysis, mitochondrial activities, and the access that retinas have in vivo to fuels. Our study focused on fuels of known importance in retina and RPE, but other metabolic pathways also need to be evaluated.^52^^,^^53^^,^^86^^–^^89^

Although the loss of capacity for metabolic flux appears to be less than the decrement in ERG responses, we do not have a way to unambiguously relate those observations. ERG a-wave and b-wave response amplitudes reflect not only the electrical activities of photoreceptors and downstream neurons, but also the overall electrical resistance between the cornea and the reference electrode placed in the body of the animal. Measurements of visual acuity using motion-tracking approaches are an effective way to evaluate visual function in the aging eye.

## Supplementary Material

Supplement 1

## References

[1] Owsley C. Aging and vision. *Vision Res*. 2011; 51(13): 1610–1622. 20974168 10.1016/j.visres.2010.10.020 PMC3049199

[2] Klein R, Klein BEK. The prevalence of age-related eye diseases and visual impairment in aging: Current estimates. *Investig Ophthalmol Vis Sci*. 2013; 54(14): ORSF5–ORSF13. 24335069 10.1167/iovs.13-12789 PMC4139275

[3] Elliott DB, Yang KCH, Whitaker D. Visual acuity changes throughout adulthood in normal, healthy eyes: Seeing beyond 6/6. *Optom Vis Sci*. 1995; 72(3): 186–191. 7609941 10.1097/00006324-199503000-00006

[4] Ferrer-Blasco T, González-Méijome JM, Montés-Micó R. Age-related changes in the human visual system and prevalence of refractive conditions in patients attending an eye clinic. *J Cataract Refract Surg*. 2008; 34(3): 424–432. 18299067 10.1016/j.jcrs.2007.10.032

[5] Gu X, Neric NJ, Crabb JS, et al. Age-related changes in the retinal pigment epithelium (RPE). *PLoS One*. 2012; 7(6): e38673. 22701690 10.1371/journal.pone.0038673 PMC3372495

[6] Bonilha VL. Age and disease-related structural changes in the retinal pigment epithelium. *Clin Ophthalmol*. 2008; 2(2): 413–424. 19668732 10.2147/opth.s2151 PMC2693982

[7] Karunadharma PP, Nordgaard CL, Olsen TW, Ferrington DA. Mitochondrial DNA damage as a potential mechanism for Age-Related macular Degeneration. *Investig Ophthalmol Vis Sci*. 2010; 51(11): 5470–5479. 20505194 10.1167/iovs.10-5429 PMC3061495

[8] Brunk UT, Terman A. Lipofuscin: Mechanisms of age-related accumulation and influence on cell function. *Free Radic Biol Med*. 2002; 33(5): 611–619. 12208347 10.1016/s0891-5849(02)00959-0

[9] Crabb JW. The proteomics of drusen. *Cold Spring Harb Perspect Med*. 2014; 4(7): a017194. 24799364 10.1101/cshperspect.a017194 PMC4066642

[10] Chen M, Rajapakse D, Fraczek M, Luo C, Forrester J V., Xu H. Retinal pigment epithelial cell multinucleation in the aging eye - a mechanism to repair damage and maintain homoeostasis. *Aging Cell*. 2016; 15: 436–445. 26875723 10.1111/acel.12447 PMC4854907

[11] Ambati J, Fowler BJ. Mechanisms of age-related macular degeneration. *Neuron*. 2012; 75(1): 26–39. 22794258 10.1016/j.neuron.2012.06.018 PMC3404137

[12] Alavi MV . Aging and Vision. In: Bowes Rickman C, LaVail MM, Anderson RE, Grimm C, Hollyfield J, Ash J, eds. *Retinal Degenerative Diseases*. Vol. 854. 1st ed. Basingstoke: Springer Nature; 2016: 393–399.

[13] Bhutto I, Lutty G. Understanding age-related macular degeneration (AMD): Relationships between the photoreceptor/retinal pigment epithelium/Bruch's membrane/choriocapillaris complex. *Mol Aspects Med*. 2012; 33(4): 295–317. 22542780 10.1016/j.mam.2012.04.005 PMC3392421

[14] Inana G, Murat C, An W, Yao X, Harris IR, Cao J. RPE phagocytic function declines in age-related macular degeneration and is rescued by human umbilical tissue derived cells. *J Transl Med*. 2018; 16: 63. 29534722 10.1186/s12967-018-1434-6 PMC5851074

[15] Curcio CA, Millican CL, Allen KA, Kalina RE. Aging of the human photoreceptor mosaic: Evidence for selective vulnerability of rods in central retina. *Investig Ophthalmol Vis Sci*. 1993; 34(12): 3278–3296. 8225863

[16] Samuel MA, Zhang Y, Meister M, Sanes JR. Age-related alterations in neurons of the mouse retina. *J Neurosci*. 2011; 31(44): 16033–16044. 22049445 10.1523/JNEUROSCI.3580-11.2011 PMC3238393

[17] Cavallotti C, Artico M, Pescosolido N, Tranquilli Leali FM, Feher J. Age-related changes in the human retina. *Can J Ophthalmol*. 2004; 39: 61–68. 15040616 10.1016/s0008-4182(04)80054-1

[18] Gresh J, Goletz PW, Crouch RK, Rohrer B. Structure-function analysis of rods and cones in juvenile, adult, and aged C57BL/6 and Balb/c mice. *Vis Neurosci*. 2003; 20(2): 211–220. 12916741 10.1017/s0952523803202108

[19] Bissig D, Goebel D, Berkowitz BA. Diminished Vision in Healthy Aging Is Associated with Increased Retinal L-Type Voltage Gated Calcium Channel Ion Influx. *PLoS One*. 2013; 8(2): e56340. 23457553 10.1371/journal.pone.0056340 PMC3572962

[20] Nag TC, Wadhwa S. Immunolocalisation pattern of complex I–V in ageing human retina: Correlation with mitochondrial ultrastructure. *Mitochondrion*. 2016; 31: 20–32. 27581213 10.1016/j.mito.2016.08.016

[21] Wang AL, Lukas TJ, Yuan M, Neufeld AH. Age-related increase in mitochondrial DNA damage and loss of DNA repair capacity in the neural retina. *Neurobiol Aging*. 2010; 31(11): 2002–2010. 19084291 10.1016/j.neurobiolaging.2008.10.019

[22] Eells JT. Mitochondrial dysfunction in the aging retina. *Biology (Basel)*. 2019; 8(2): 31. 10.3390/biology8020031 PMC6627398 31083549

[23] Wang Y, Grenell A, Zhong F, et al. Metabolic signature of the aging eye in mice. *Neurobiol Aging*. 2018; 71: 223–233. 30172221 10.1016/j.neurobiolaging.2018.07.024 PMC6162115

[24] Kolesnikov A V., Fan J, Crouch RK, Kefalov VJ. Age-related deterioration of rod vision in mice. *J Neurosci*. 2010; 30(33): 11222–11231. 20720130 10.1523/JNEUROSCI.4239-09.2010 PMC2928554

[25] Freund PR, Watson J, Gilmour GS, Gaillard F, Sauvé Y. Differential changes in retina function with normal aging in humans. *Doc Ophthalmol*. 2011; 122: 177–190. 21562738 10.1007/s10633-011-9273-2

[26] Kergoat H, Kergoat M-J, Justino L. Age-related changes in the flash electroretinogram and oscillatory potentials in individuals age 75 and older. *J Am Geriatr Soc*. 2001; 49: 1212–1217. 11559381 10.1046/j.1532-5415.2001.49239.x

[27] Berkowitz BA, Grady EM, Roberts R. Confirming a prediction of the calcium hypothesis of photoreceptor aging in mice. *Neurobiol Aging*. 2014; 35(8): 1883–1891. 24680323 10.1016/j.neurobiolaging.2014.02.020

[28] Lehmann K, Schmidt KF, Löwel S. Vision and visual plasticity in ageing mice. *Restor Neurol Neurosci*. 2012; 30(2): 161–178. 22348872 10.3233/RNN-2012-110192

[29] Vit JP, Fuchs DT, Angel A, et al. Color and contrast vision in mouse models of aging and Alzheimer's disease using a novel visual-stimuli four-arm maze. *Sci Rep*. 2021; 11(1): 1–19. 33441984 10.1038/s41598-021-80988-0 PMC7806734

[30] Feng L, Sun Z, Han H, Zhou Y, Zhang M. No age-related cell loss in three retinal nuclear layers of the Long-Evans rat. *Vis Neurosci*. 2007; 24(6): 799–803. 18093367 10.1017/S0952523807070721

[31] Fulton AB, Dodge J, Hansen RM, Williams TP. The Rhodopsin Content of Human Eyes. *Invest Ophthalmol Vis Sci*. 1999; 40(8): 1878–1883. 10393065

[32] Winkler BS. Glycolytic and oxidative metabolism in relation to retinal function. *J Gen Physiol*. 1981; 77(6): 667–692. 6267165 10.1085/jgp.77.6.667 PMC2215447

[33] Kanow MA, Giarmarco MM, Jankowski C, et al. Biochemical adaptations of the retina and retinal pigment epithelium support a metabolic ecosystem in the vertebrate eye. *Elife*. 2017; 6: e28899. 28901286 10.7554/eLife.28899 PMC5617631

[34] Haydinger CD, Kittipassorn T, Peet DJ. Power to see—Drivers of aerobic glycolysis in the mammalian retina: A review. *Clin Exp Ophthalmol*. 2020; 48: 1057–1071. 32710505 10.1111/ceo.13833

[35] Warburg O. The metabolism of carcinoma cells. *J Cancer Res*. 1925; 9(1): 148–163.

[36] Swarup A, Samuels IS, Bell BA, et al. Modulating GLUT1 expression in the RPE decreases glucose levels in the retina: Impact on photoreceptors and Müller glial cells. *Am J Physiol Physiol*. 2019; 316(1): C121–C133. 10.1152/ajpcell.00410.2018 PMC6383144 30462537

[37] Kurihara T, Westenskow PD, Gantner ML, et al. Hypoxia-induced metabolic stress in retinal pigment epithelial cells is sufficient to induce photoreceptor degeneration. *Elife*. 2016; 5: e14319. 26978795 10.7554/eLife.14319 PMC4848091

[38] Zhao C, Yasumura D, Li X, et al. mTOR-mediated dedifferentiation of the retinal pigment epithelium initiates photoreceptor degeneration in mice. *J Clin Invest*. 2011; 121(1): 369–383. 21135502 10.1172/JCI44303 PMC3007156

[39] Du J, Linton JD, Hurley JB. Probing Metabolism in the Intact Retina Using Stable Isotope Tracers Jianhai. *Methods Enzymol*. 2015; 561: 149–170. 26358904 10.1016/bs.mie.2015.04.002 PMC6261279

[40] Mattapallil MJ, Wawrousek EF, Chan CC, et al. The Rd8 mutation of the Crb1 gene is present in vendor lines of C57BL/6N mice and embryonic stem cells, and confounds ocular induced mutant phenotypes. *Invest Ophthalmol Vis Sci*. 2012; 53(6): 2921–2927. 22447858 10.1167/iovs.12-9662 PMC3376073

[41] Chang B, Hurd R, Wang J, Nishina P. Survey of Common Eye Diseases in Laboratory Mouse Strains. *Investig Ophthalmol Vis Sci*. 2013; 54(7): 4974–4981. 23800770 10.1167/iovs.13-12289 PMC3723375

[42] Millard P, Letisse F, Sokol S, Portais JC. IsoCor: correcting MS data in isotope labeling experiments. *Bioinformatics*. 2012; 28(9): 1294–1296. 22419781 10.1093/bioinformatics/bts127

[43] Millard P, Delépine B, Guionnet M, Heuillet M, Bellvert F, Létisse F. IsoCor: isotope correction for high-resolution MS labeling experiments. *Bioinformatics*. 2019; 35(21): 4484–4487. 30903185 10.1093/bioinformatics/btz209

[44] Sweet IR, Cook DL, Wiseman RW, et al. Dynamic perifusion to maintain and assess isolated pancreatic islets. *Diabetes Technol Ther*. 2002; 4(1): 67–76. 12017423 10.1089/15209150252924111

[45] Sweet IR, Khalil G, Wallen AR, et al. Continuous Measurement of Oxygen Consumption by Pancreatic Islets. *Diabetes Technol Ther*. 2002; 4(5): 661–672. 12450449 10.1089/152091502320798303

[46] Yang N-C, Ho W, Chen Y-H, Hu M-L. A convenient one-step extraction of cellular ATP using boiling water for the luciferin-luciferase assay of ATP. *Anal Biochem*. 2002; 306(2): 323–327. 12123672 10.1006/abio.2002.5698

[47] Karp NA, Mason J, Beaudet AL, et al. Prevalence of sexual dimorphism in mammalian phenotypic traits. *Nat Commun*. 2017; 8: 15475. 28650954 10.1038/ncomms15475 PMC5490203

[48] Shaqiri A, Roinishvili M, Grzeczkowski L, et al. Sex-related differences in vision are heterogeneous. *Sci Rep*. 2018; 8: 7521. 29760400 10.1038/s41598-018-25298-8 PMC5951855

[49] Zetterberg M. Age-related eye disease and gender. *Maturitas*. 2016; 83: 19–26. 26508081 10.1016/j.maturitas.2015.10.005

[50] Du M, Mangold CA, Bixler G V., et al. Retinal gene expression responses to aging are sexually divergent. *Mol Vis*. 2017; 23: 707–717. 29062222 PMC5640516

[51] López-Otín C, Blasco MA, Partridge L, Serrano M, Kroemer G. The hallmarks of aging. *Cell*. 2013; 153(6): 1194–1217. 23746838 10.1016/j.cell.2013.05.039 PMC3836174

[52] Bisbach CM, Hass DT, Robbings BM, et al. Succinate Can Shuttle Reducing Power from the Hypoxic Retina to the O2-Rich Pigment Epithelium. *Cell Rep*. 2020; 31(5): 107606. 32375026 10.1016/j.celrep.2020.107606 PMC7273505

[53] Du J, Yanagida A, Knight K, et al. Reductive carboxylation is a major metabolic pathway in the retinal pigment epithelium. *Proc Natl Acad Sci USA*. 2016; 113(51): 14710–14715. 27911769 10.1073/pnas.1604572113 PMC5187684

[54] Yuan R, Tsaih S-W, Petkova SB, et al. Aging in inbred strains of mice: Study design and interim report on median lifespans and circulating IGF1 levels. *Aging Cell*. 2009; 8(3): 277–287. 19627267 10.1111/j.1474-9726.2009.00478.x PMC2768517

[55] Yuan R, Peters LL, Paigen B. Mice as a mammalian model for research on the genetics of aging. *ILAR J*. 2011; 52(1): 4–15. 21411853 10.1093/ilar.52.1.4 PMC3074346

[56] The Jackson Laboratory. Life span as a biomarker, https://www.jax.org/research-and-faculty/research-labs/the-harrison-lab/gerontology/life-span-as-a-biomarker. Accessed April 4, 2021.

[57] Hamberger AC, Chiang GH, Nylén ES, Scheff SW, Cotman CW. Glutamate as a CNS transmitter. I. Evaluation of glucose and glutamine as precursors for the synthesis of preferentially released glutamate. *Brain Res*. 1979; 168(3): 513–530. 435980 10.1016/0006-8993(79)90306-8

[58] Tait SWG, Green DR. Mitochondria and cell signalling. *J Cell Sci*. 2012; 125: 807–815. 22448037 10.1242/jcs.099234 PMC3311926

[59] Abate M, Festa A, Falco M, et al. Mitochondria as playmakers of apoptosis, autophagy and senescence. *Semin Cell Dev Biol*. 2020; 98: 139–153. 31154010 10.1016/j.semcdb.2019.05.022

[60] Hoppins S. The regulation of mitochondrial dynamics. *Curr Opin Cell Biol*. 2014; 29: 46–52. 24747170 10.1016/j.ceb.2014.03.005

[61] Bénit P, Letouzé E, Rak M, et al. Unsuspected task for an old team: Succinate, fumarate and other Krebs cycle acids in metabolic remodeling. *Biochim Biophys Acta - Bioenerg*. 2014; 1837(8): 1330–1337. 10.1016/j.bbabio.2014.03.013 24699309

[62] Salminen A, Kauppinen A, Hiltunen M, Kaarniranta K. Krebs cycle intermediates regulate DNA and histone methylation: Epigenetic impact on the aging process. *Ageing Res Rev*. 2014; 16: 45–65. 24910305 10.1016/j.arr.2014.05.004

[63] Wiley CD, Velarde MC, Lecot P, et al. Mitochondrial dysfunction induces senescence with a distinct secretory phenotype. *Cell Metab*. 2016; 23(2): 303–314. 26686024 10.1016/j.cmet.2015.11.011 PMC4749409

[64] Bratic A, Larsson NG. The role of mitochondria in aging. *J Clin Invest*. 2013; 123(3): 951–957. 23454757 10.1172/JCI64125 PMC3582127

[65] Bratic I, Trifunovic A. Mitochondrial energy metabolism and ageing. *Biochim Biophys Acta - Bioenerg*. 2010; 1797(6-7): 961–967. 10.1016/j.bbabio.2010.01.004 20064485

[66] Sun N, Youle RJ, Finkel T. The Mitochondrial Basis of Aging. *Mol Cell*. 2016; 61(5): 654–666. 26942670 10.1016/j.molcel.2016.01.028 PMC4779179

[67] Camandola S, Mattson MP. Brain metabolism in health, aging, and neurodegeneration. *EMBO J*. 2017; 36(11): 1474–1492. 28438892 10.15252/embj.201695810 PMC5452017

[68] Angelin A, Gil-de-Gómez L, Dahiya S, et al. Foxp3 Reprograms T Cell Metabolism to Function in Low-Glucose, High-Lactate Environments. *Cell Metab*. 2017; 25(6): 1282–1293. 28416194 10.1016/j.cmet.2016.12.018 PMC5462872

[69] Goyal MS, Vlassenko AG, Blazey TM, et al. Loss of Brain Aerobic Glycolysis in Normal Human Aging. *Cell Metab*. 2017; 26(2): 353–360. 28768174 10.1016/j.cmet.2017.07.010 PMC5573225

[70] Weyand CM, Goronzy JJ. Aging of the immune system: Mechanisms and therapeutic targets. *Ann Am Thorac Soc*. 2016; 13: S422–S428. 28005419 10.1513/AnnalsATS.201602-095AW PMC5291468

[71] Ritterhoff J, Tian R. Metabolismin cardiomyopathy: Every substrate matters. *Cardiovasc Res*. 2017; 113(4): 411–421. 28395011 10.1093/cvr/cvx017 PMC5852620

[72] Chiao YA, Rabinovitch PS. The aging heart. *Cold Spring Harb Perspect Med*. 2015; 5(9): a025148. 26328932 10.1101/cshperspect.a025148 PMC4561390

[73] Rui L. Energy Metabolism in the Liver. *Compr Physiol*. 2014; 4(1): 177–197. 24692138 10.1002/cphy.c130024 PMC4050641

[74] Vitorica J, Satrustegui A, Machado A. Metabolic Implications of Ageing: Changes in Activities of Key Lipogenic and Gluconeogenic Enzymes in the Aged Rat Liver. *Enzyme*. 1981; 26: 144–152. 6265205 10.1159/000459164

[75] Garvey SM, Dugle JE, Kennedy AD, et al. Metabolomic profiling reveals severe skeletal muscle group-specific perturbations of metabolism in aged FBN rats. *Biogerontology*. 2014; 15: 217–232. 24652515 10.1007/s10522-014-9492-5 PMC4019835

[76] Nishikawa T, Bellancce N, Damm A, et al. A switch in the source of ATP production and a loss in capacity to perform glycolysis are hallmarks of hepatocyte failure in advance liver disease. *J Hepatol*. 2014; 60(6): 1203–1211. 24583248 10.1016/j.jhep.2014.02.014 PMC4028384

[77] Demontis F, Piccirillo R, Goldberg AL, Perrimon N. Mechanisms of skeletal muscle aging: Insights from Drosophila and mammalian models. *DMM Dis Model Mech*. 2013; 6: 1339–1352. 24092876 10.1242/dmm.012559 PMC3820258

[78] Ohlendieck K. Proteomic profiling of fast-to-slow muscle transitions during aging. *Front Physiol*. 2011; 2: 105. 22207852 10.3389/fphys.2011.00105 PMC3245893

[79] Wei Y, Jiang H, Shi Y, et al. Age-related alterations in the retinal microvasculature, microcirculation, and microstructure. *Investig Ophthalmol Vis Sci*. 2017; 58(9): 3804–3817. 28744554 10.1167/iovs.17-21460 PMC5527847

[80] Lin Y, Jiang H, Liu Y, et al. Age-related alterations in retinal tissue perfusion and volumetric vessel density. *Investig Ophthalmol Vis Sci*. 2019; 60(2): 685–693. 30786280 10.1167/iovs.18-25864 PMC6383727

[81] Orlov N V., Coletta C, van Asten F, et al. Age-related changes of the retinal microvasculature. *PLoS One*. 2019; 14(5): e0215916. 31048908 10.1371/journal.pone.0215916 PMC6497255

[82] Bata AM, Fondi K, Szegedi S, et al. Age-Related Decline of Retinal Oxygen Extraction in Healthy Subjects. *Investig Ophthalmol Vis Sci*. 2019; 60(8): 3162–3169. 31335953 10.1167/iovs.18-26234

[83] Hutto RA, Bisbach CM, Abbas F, et al. Increasing Ca2+ in photoreceptor mitochondria alters metabolites, accelerates photo response recovery, and reveals adaptations to mitochondrial stress. *Cell Death Differ*. 2020; 27: 1067–1085. 31371786 10.1038/s41418-019-0398-2 PMC7206026

[84] Grenell A, Wang Y, Yam M, et al. Loss of MPC1 reprograms retinal metabolism to impair visual function. *Proc Natl Acad Sci USA*. 2019; 116(9): 3530–3535. 30808746 10.1073/pnas.1812941116 PMC6397593

[85] TeSlaa T, Bartman CR, Jankowski CSR, et al. The Source of Glycolytic Intermediates in Mammalian Tissues. *Cell Metab*. 2021; 33(2): 367–378. 33472024 10.1016/j.cmet.2020.12.020 PMC8088818

[86] Adijanto J, Du J, Moffat C, Seifert EL, Hurley JB, Philp NJ. The retinal pigment epithelium utilizes fatty acids for ketogenesis. *J Biol Chem*. 2014; 289(30): 20570–20582. 24898254 10.1074/jbc.M114.565457 PMC4110270

[87] Reyes-Reveles J, Dhingra A, Alexander D, Bragin A, Philp NJ, Boesze-Battaglia K. Phagocytosis-dependent ketogenesis in retinal pigment epithelium. *J Biol Chem*. 2017; 292(19): 8038–8047. 28302729 10.1074/jbc.M116.770784 PMC5427279

[88] Izuta Y, Imada T, Hisamura R, et al. Ketone body 3-hydroxybutyrate mimics calorie restriction via the Nrf2 activator, fumarate, in the retina. *Aging Cell*. 2017; 17(1): e12699. 10.1111/acel.12699 PMC5770878 29119686

[89] Yam M, Engel AL, Wang Y, et al. Proline mediates metabolic communication between retinal pigment epithelial cells and the retina. *J Biol Chem*. 2019; 294(26): P10278–P10289. 10.1074/jbc.RA119.007983 PMC6664195 31110046

